# Detection of Parvovirus B19 genome in human heart tissue samples

**DOI:** 10.1186/s13104-023-06527-4

**Published:** 2023-09-29

**Authors:** Anna Kloc, Kenneth S. Campbell, Yarida A. Urbina Espinoza

**Affiliations:** 1 Department of Biology and Environmental Science, University of New Haven, West Haven, CT USA; 2 Saha Cardiovascular Research Center, University of Kentucky College of Medicine, Lexington, KY USA

**Keywords:** Parvovirus B19, Viral persistence, Heart disease, Virus identification

## Abstract

**Objective:**

Identifying viral genomes in human heart tissues is critical for disease diagnosis and assessment of cardiovascular damage. Human heart tissue samples obtained during a biopsy procedure are routinely used to test for the presence of viruses, as guided by clinical manifestations and prognosis. Furthermore, heart tissue samples obtained post-mortem or during a cardiac transplant procedure serve as a valuable research tool, as they allow for an in-depth assessment of cardiac pathology that can aid in our understanding of molecular pathways associated with disease. Because viral nucleic acid constitutes only a small portion of each sample’s genetic material, appropriate methods are necessary for positive viral genome identification.

**Results:**

Snap-frozen heart tissue samples obtained either post-mortem or during a cardiac transplant procedure were used to develop conditions for detection of Parvovirus B19. Briefly, total DNA was isolated from the heart tissue under varying conditions. A PCR-based assay with Parvovirus B19 specific primers was implemented to detect the presence of the viral genome, followed by Sanger Sequencing. The mechanical disruption of the heart tissue, as well as the cardiac tissue processing methods, had a significant effect on the DNA quality and the ability to detect the Parvovirus B19 genome.

## Introduction

Inflammation of the cardiac muscle, or myocarditis, is a well-known phenomenon that affects approximately 1.5 million individuals each year [1]. The disease is most commonly associated with viral infection although pathogens, such as bacteria or parasites, have also been reported as a cause [2, 3]. Endomyocardial biopsy (EMB) combined with immunohistochemistry analysis are critical for making a clinical diagnosis, as EMB assesses the presence of a pathogen, and immunohistochemistry reveals pathological features, such as cellular infiltrates and necrotic tissue [4, 5]. Virus-induced acute cardiac inflammation is known to cause cardiac distress, and severe cases of heart inflammation can result in patient’s death. If the virus is not eliminated from the host, it can persist in the cardiac tissue for an extended period of time, often leading to cardiomyocyte injury and cardiac muscle remodeling [6]. Previous studies have shown that Parvovirus B19, adenovirus, coxsackievirus B3, Epstein-Barr virus, human cytomegalovirus and human herpesvirus 6 are most commonly found in the human heart tissue samples [7–9]. While direct cardiomyocyte damage that is a result of viral infection can impair the heart, viral persistence in cells commonly found in the heart has been suggested to contribute to, or exacerbate, cardiac symptoms.

Parvovirus B19 (B19V), one of the most prevalent viruses found in the human cardiac tissue during an EMB procedure, is associated with a common childhood infection, known as erythema infectiosum [10]. B19V has been shown to cause acute myocarditis in the past [11, 12]. Even though the virus does not directly infect cardiomyocytes, the cardiac damage is thought to occur via infection of endothelial cells of the myocardial vessels and activation of inflammatory cells [13–15]. The persistence of B19V in the cardiac tissue after the initial infection is a well-documented phenomenon that has been associated with atypical angina pectoris [16] and mitochondrial impairment [17]. Yet, the mechanism that links the long-term viral presence to negative cardiovascular outcomes is not fully understood, especially considering that the virus has been found in healthy individuals [18]. To better understand the potential relationship between cardiac damage and B19V persistence in the context of cardiovascular health, we need reliable techniques that allow for identification of low-copy B19V genomes in the human cardiac tissue.

Currently, obtaining an EMB sample from a patient’s heart, followed by positive identification of viral genome or protein, is the only method that can definitively link cardiac inflammation to viral infection. Human heart tissue samples can also be used for biological research to discover mechanisms that underlie virus-induced damage to the heart. These heart tissue samples are processed to obtain DNA and RNA, and the presence of viral genome(s) is commonly assessed using a PCR-based analysis with primers specific to particular viral genome regions [19]. The successful identification of a viral genome depends on the amount of initial EMB sample, the quality of extracted DNA/RNA, and the successful PCR amplification. An acute heart inflammation caused by a viral infection is likely accompanied by high virus quantity, which can be readily assessed in a PCR reaction. However, when dealing with a persistent viral infection where the overall amount of the virus may be lower, multiple rounds of DNA/RNA extraction may be needed to successfully identify viral genomes. Here, we tested experimental conditions for extraction of DNA from human heart tissue samples and subsequent amplification of the B19V genome. We show that this method can be used to successfully identify the B19V genome in varying amounts of human heart tissues.

## Methods

### Heart tissue samples

Human heart tissues were obtained from the UK Gill Heart & Vascular Institute, KY. The tissues obtained using a previously described protocol [20] were derived from heart transplants or ventricular assist device (VAD) implants, and flash frozen within 30 min of being removed from the patient. The human heart contains three layers: epicardium, endocardium and myocardium. All three layers can be processed to obtain DNA/RNA, and test for the presence of B19V, using the protocol described below. In lab, an individual layer was dissected to yield 25 mg ± 2 mg, 50 mg ± 2 mg or 100 mg ± 2 mg samples right before the nucleic acid isolation procedure.

### DNA extraction

Dissected heart tissues (25 mg ± 2 mg, 50 mg ± 2 mg or 100 mg ± 2 mg) were homogenized using a PRO Scientific Bio-Gen homogenizer, and the dissociated tissues were incubated for either 1 h, 2 h, 4 h or 24 h at 55 °C in 1 × Shield Buffer, and in the presence of 25 µl of proteinase K (20 mg/ml). Simultaneously, non-homogenized heart tissues (25 mg ± 2 mg, 50 mg ± 2 mg or 100 mg ± 2 mg) were incubated in 1 × Shield Buffer for either 1 h, 2 h, 4 h or 24 h at 55 °C in the presence of 25 µl of proteinase K (20 mg/ml). Homogenized and non-homogenized samples were then processed to isolate the nucleic acid using a Quick-DNA/RNA Miniprep Plus Kit (Zymo Research, D7003). NanoDrop One spectrophotometry (Thermo Scientific) was used to quantify the obtained DNA, and the DNA integrity was visually assessed on a 1% agarose gel.

### B19V genome and human β-actin gene amplification

PCR primers specific to the B19V NS1 gene (NS1F 5ʹ TTGTCAAAACTATGACCCCCTATT 3ʹ and NS1R 5ʹ TAACCATGCCATATACTGGAACAC 3ʹ) were used in a PCR reaction in the presence of Phusion DNA Polymerase (Phusion High-Fidelity PCR Kit, ThermoFisher) to yield a 146 bp fragment. We used a human β-actin (ACTB) gene (ACTBF 5ʹ CCTGCAGAGTTCCAAAGGAG 3ʹ and ACTBR 5ʹ AAGATGACCCAGGTGAGTGG 3ʹ) as a control for human genome amplification to yield a 135 bp fragment. The amplified B19V NS1 and human ACTB gene fragments were visualized using 1% agarose gel electrophoresis. The B19V NS1 PCR products were sequenced using Sanger Sequencing to verify the NS1 gene identity.

## Results

### Nucleic acid extraction

**Fig. 1:**
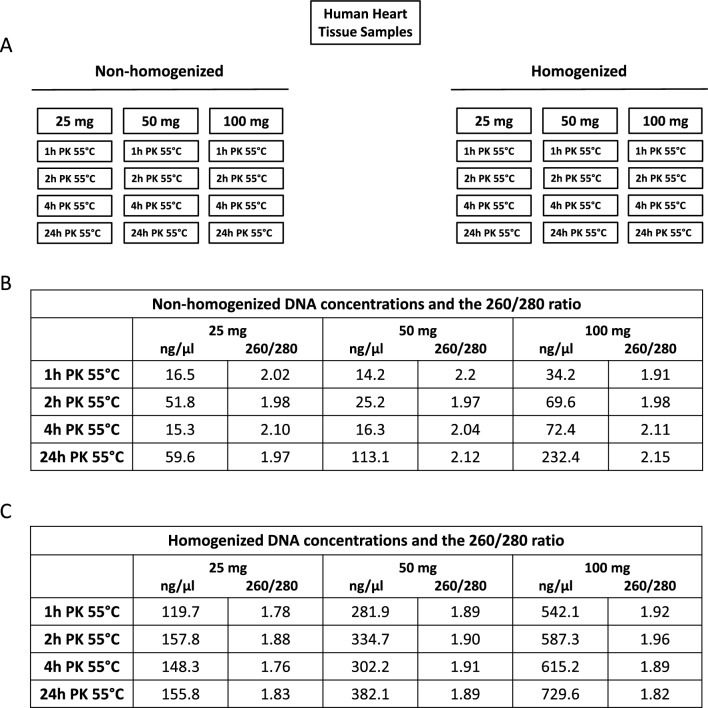
Assessment of DNA isolated from non-homogenized and homogenized human heart tissue samples. **A** Image of experimental scheme with specific DNA amounts and proteinase K (PK) treatment times. **B** Quantification (ng/µl) and absorbance 260/280 ratio of non-homogenized DNA samples. **C** Quantification (ng/µl) and absorbance 260/280 ratio of homogenized DNA samples

Isolating viral genetic material from human heart clinical samples is often challenging due to limited material availability. The human heart tissue is hard to dissociate, which could affect the overall yield of both human and viral nucleic acid during an isolation procedure. Mechanical dissociation of the heart tissue is often combined with the nucleic acid extraction methods, as it can impact the quality of the obtained nucleic acid. Furthermore, the viral genetic material constitutes only a small fraction of the total DNA/RNA material, which may impact the identification and characterization of low-copy viral genomes that may be present in the heart tissue. Therefore, obtaining sufficient amounts of viral genetic material is critical for the subsequent PCR identification. Here, we tested increasing amounts of frozen human heart tissue for B19V genetic material isolation under variable conditions (Fig. 1A). Briefly, 25 mg ± 2 mg, 50 mg ± 2 mg or 100 mg ± 2 mg of the human heart tissue was homogenized for 15–20 s at medium speed to achieve sample dissociation. The samples were incubated in a 55 °C water bath in the presence of proteinase K (20 mg/ml) for either 1, 2, 4 or 24 h. Concurrently, non-homogenized human heart tissue samples corresponding to 25 mg ± 2 mg, 50 mg ± 2 mg or 100 mg ± 2 mg were incubated in a 55 °C water bath in the presence of proteinase K (20 mg/ml) for either 1, 2, 4 or 24 h.

### Assessment of DNA quantity

Total DNA concentrations of all processed DNA samples were measured using a NanoDrop One (Fig. 1B, C). The highest amounts of total DNA were recovered in homogenized samples treated with proteinase K, whereas the lack of heart tissue dissociation prior to nucleic acid extraction negatively impacted the amounts of isolated DNA. The length of incubation with proteinase K also impacted the amounts of total recovered DNA regardless of previous heart tissue dissociated status. Specifically, the highest amount of total DNA was obtained from both homogenized and non-homogenized heart tissues incubated with proteinase K for 24 h at 55 °C.

### Identification of B19V genome from extracted heart tissue samples

**Fig. 2:**
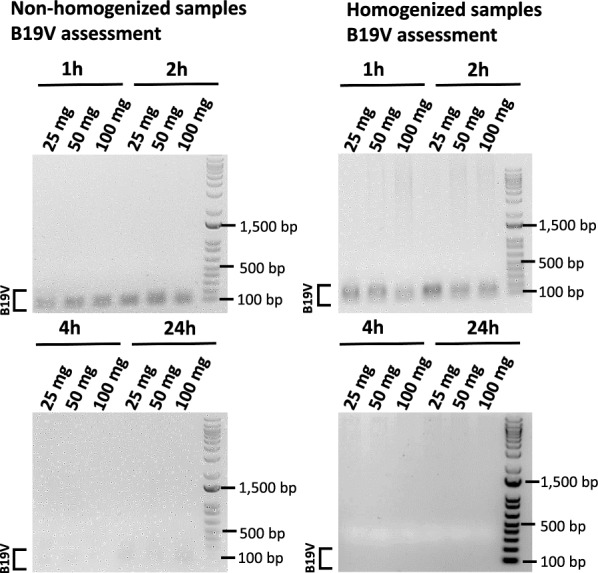
Amplification of NS1 (B19V) in extracted DNA samples. Homogenized and non-homogenized heart tissues were incubated at 55 °C in the presence of proteinase K for 1, 2, 4 and 24 h. PCR products (146 bp) were visualized using 1% agarose gel electrophoresis

2 µl of total DNA from each individual extraction procedure was used in the presence of the B19V NS1 primers in a PCR reaction. Agarose gel electrophoresis of the PCR-amplified DNA revealed a 146 bp band in all non-homogenized samples (25 mg ± 2 mg, 50 mg ± 2 mg or 100 mg ± 2 mg) that were incubated in the presence of proteinase K for 1 and 2 h only (Fig. 2). No PCR product was obtained when the non-homogenized samples were incubated with proteinase K for 4 or 24 h. Similarly, a 146 bp band corresponding to the B19V NS1 gene was visualized in previously homogenized samples incubated with proteinase K for either 1 or 2 h at 55 °C. No PCR band was visualized in samples incubated with proteinase K for 4, or 24 h at 55 °C (Fig. 2).

### Assessment of human β-actin (ACTB) amplification

**Fig. 3:**
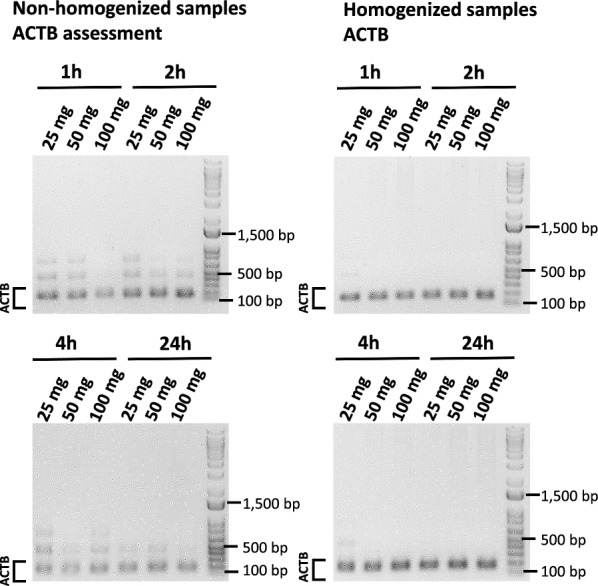
Amplification of ACTB in extracted DNA samples. Homogenized and non-homogenized heart tissues were incubated at 55 °C in the presence of proteinase K for 1, 2, 4 and 24 h. PCR products (135 bp) were visualized using 1% agarose gel electrophoresis

The human ACTB gene was amplified in all processed samples regardless of the amount of the starting heart tissue material and the sample dissociation status. However, the PCR reactions of non-homogenized DNA samples produced numerous non-specific bands (Fig. 3). On the contrary, homogenizing the heart tissues prior to incubation with proteinase K improved the specificity of the PCR reaction (Fig. 3).

## Discussion

Viral persistence in human heart tissue is a well-recognized phenomenon. The identification of viruses in the human heart is important in clinical setting, but also for scientific research purposes. Therefore, the isolation of genetic material from the heart tissue is critical for successful identification of these viruses. Processing of the heart tissue often requires mechanical dissociation and long incubation periods in the presence of proteinase K. These factors can impact the likelihood of isolating viral genetic material present in low quantities.

Here, we utilized frozen heart tissues to produce an efficient method to identify B19V viral genome under varying experimental conditions. We observed that incubating non-homogenized heart tissues with proteinase K (20 mg/ml) for either 1 or 2 h resulted in positive B19V identification despite low yields of total DNA (Fig. 2). Specifically, we were able to amplify the B19V NS1 gene in a PCR reaction, and confirm its identity with Sanger Sequencing. Prolonged incubation of the heart tissue samples with proteinase K (4, and 24 h) improved the overall DNA yield at the expense of viral DNA identification. In fact, no B19V DNA was amplified when the heart tissues were incubated for either 4 or 24 h at 55 °C, which is likely due to viral DNA degradation at these experimental conditions (Fig. 2).

Dissociation of the heart tissue with a homogenizer prior to incubation with proteinase K at 55 °C yielded higher amounts of total DNA and resulted in better DNA quality (Figs. 1&3). Specifically, homogenizing the heart tissue yielded a 260/280 ratio close to 1.8–2, which is indicative of DNA purity. Non-homogenized tissue tends to have a 260/280 ratio that is higher than 2, which suggests RNA contamination. Amplification with ACTB specific primers yielded a more specific amplicon if the heart tissue was mechanically dissociated prior to the nucleic acid procedure, suggesting that homogenization improves the overall DNA quality. However, even though the homogenized samples that had a higher nucleic acid yield, the B19V DNA could only be recovered if the DNA samples were incubated with proteinase K at 55 °C for either 1 or 2 h (Fig. 3). In sum, both the recovery of the genetic material from the heart tissues, and the subsequent identification of the B19V NS1 gene was optimal when the samples were homogenized prior to proteinase K (20 mg/ml) treatment, and incubated with the enzyme at 55 °C for up to 2 h post-dissociation. These results indicate that low-copy viral genomes that may be present in human heart tissue samples are prone to degradation during the nucleic acid extraction procedure, which may impact the likelihood of positive B19V virus identification.

## Limitations

Scientific replicas of individual time points obtained from the same heart tissue sample were not performed due to limited material availability. Instead, a set of 3 separate heart tissue samples derived from other individuals was processed according to the described experimental scheme with equivalent results (data not shown).

## Data Availability

The data generated during this study is included in the published article.

## Abbreviations

B19V: Parvovirus B19
ACTB: Human β-actin
